# Association between Alcohol Use Disorder and Suicidal Ideation Using Propensity Score Matching in Chungcheongnam-do, South Korea

**DOI:** 10.3390/healthcare12131315

**Published:** 2024-06-30

**Authors:** Jeong-Min Yang, Jae-Hyun Kim, Min-Soo Kim, Ji-Sung Hong, Bon-Hee Gu, Ju-Ho Park, Young-Long Choi, Jung-Jae Lee

**Affiliations:** 1 Department of Public Health, General Graduate School, Dankook University, Cheonan 31116, Republic of Korea; yangjm1206@gmail.com (J.-M.Y.); soo931108@gmail.com (M.-S.K.); entlfch@dankook.ac.kr (J.-S.H.); 9qhsgml@gmail.com (B.-H.G.); skypillar1092@dankook.ac.kr (J.-H.P.); younglong@dankook.ac.kr (Y.-L.C.); 2 Department of Health Administration, College of Health Science, Dankook University, Cheonan 31116, Republic of Korea; j_mini97@naver.com; 3 Department of Psychiatry, College of Medicine, Dankook University, Cheonan 31116, Republic of Korea

**Keywords:** alcohol use disorder, propensity score matching, suicidal ideation

## Abstract

**Objectives:** This study aimed to analyze the association between alcohol use disorder (AUD) and suicidal ideation (SI) in the general Korean population. **Methods:** The 2022 Mental Health Awareness Survey was collected from the Chungcheongnam-do Mental Health Welfare Center (CHMHC). Before Propensity Score Matching (PSM), 823 participants were included in this study. After 1:4 PSM, the 255 participants were analyzed using the chi-square test and matched conditional logistic regression. **Results:** The AUD group had higher odds of experiencing SI than the non-AUD (adjusted odds ratio [AOR]: 2.40, 95% confidence intervals [CI]: 1.10–5.22). Stratified matched conditional logistic regression showed that, among the female, <40 years and single group, the AUD group was more likely to experience SI compared with the non-AUD, respectively (AOR:3.53, 95% CI: 1.20–10.44/AOR:3.45, 95% CI: 1.03–11.55/AOR:4.83, 95% CI: 1.18–19.69). However, among the male, ≥40 years and married group, we discovered no association between AUD and SI. **Conclusions:** Through this study, we found a strong association between the AUD group and SI. This association was particularly strong among female, <40 years, and single groups. This study elucidates the relationship between AUD and SI in the Chungnam region, which had not been previously identified in Korea, and it is expected to serve as foundational data for reducing the high suicide rate in this region. However, due to the limitation of being a cross-sectional study, future longitudinal research is required.

## 1. Introduction

Alcohol is not only abused worldwide as a harmful substance that affects the body, including the brain, but also is one of the leading causes of preventable death [1]. According to the World Health Organization (WHO) [2], the number of deaths caused by alcohol consumption is 3 million annually, accounting for 5.3% of all deaths, and it has been identified as the fifth leading risk factor for disease burden [3]. In addition, most of the people who suffer from alcohol-related diseases and deaths have a high prevalence of alcohol use disorder (AUD) [1]. According to the DSM-5, AUD is the most common mental disorder worldwide, and it is defined as having a higher prevalence of alcohol consumption than is recommended [4]. AUD is characterized by compulsive excessive alcohol use and a loss of control over alcohol consumption. It is one of the most prevalent mental disorders globally, particularly in high-income and upper-middle-income countries, and it is primarily associated with high mortality rates and disease burden due to medical consequences such as liver cirrhosis or injuries [5].

However, according to the OECD published data [6], which provide international statistics for comparing pure alcohol consumption across countries, despite the fact that South Korea’s pure alcohol consumption as of 2022 is relatively low compared to the OECD average of 8.4 L, the United States (U.S) at 9.3 L, and France at 10.4 L, with an annual consumption of 7.9 L, the prevalence of AUD in South Korea is quite high at 11.6% compared to the U.S [7,8]. In the case of South Korea, it has a unique culture where social and business relationships are often maintained through alcohol consumption, which can lead to high-risk drinking habits such as binge drinking and heavy drinking compared to other countries [9,10,11]. According to the WHO, South Korea’s high-risk drinking rate is 14.9%, which is significantly higher than the global average (11.5%), the U.S (8.2%), and France (5.3%), making it highly vulnerable to AUD [12].

Due to the characteristic loss of control over compulsive drinking and alcohol consumption, AUD not only causes physical problems, such as alcoholic liver disease [5], but also leads to long-term economic difficulties, such as employment changes, as well as mental disorders, such as sleep disorders, depression, anxiety disorders, and even suicidal ideation (SI) [13,14]. In addition, AUD has been reported to increase the risk of suicide as an independent risk factor by causing disorders in the endocrine system and other factors, not just as a complex mediator with other mental disorders [15].

However, according to the WHO report [16], about 80% of the group with SI do not progress to a suicide attempt, as they often only have a temporary suicidal impulse [17]. Nevertheless, SI is considered a crucial primary indicator of the potential for a suicide attempt, as it is an essential stage in the process of suicide [18]. In fact, the SI rate in one of Korea’s administrative districts, “Chungcheongnam-do (Chungnam)”, was reported to be 7.5%, which is considered high within Korea [19]. Similarly, the suicide rate per 100,000 population in the Chungnam region was also reported to be 45.0, which is not only the highest in Korea but also significantly higher than the OECD average [20]. The Chungnam region has been classified as a vulnerable area for AUD due to its consistently high high-risk drinking rates over the past 10 years [21], and it has been reported that alcohol problems are a major cause of SI [22]. The high rate of risk drinking is generally attributed to the high drinking rates among younger age groups and low levels of health awareness [23]. However, the specific causes of the high drinking rates in Chungcheongnam-do remain unidentified to date. In addition, a previous study in China reported that the SI rate was higher in the AUD group than in the non-AUD group [24]. Another previous study in Japan, which targeted 770 participants, also found that the SI rates for men and women with AUD were 15.4% and 4.9% higher, respectively, than those in the control group [25].

As a result of these issues, Korean health authorities have been implementing various alcohol prevention and suicide prevention programs targeting high-risk drinkers to reduce the high suicide risk associated with AUD [26]. However, due to the characteristic of AUD not manifesting physical and mental symptoms until it reaches a severe stage, as well the lack of detailed policies to manage it, the current treatment rate for AUD in Korea is 6.5%, which is lower than the 11.8% in the U.S [27]. In addition, although studies investigating the association between AUD and SI or attempts are being conducted in foreign countries, there is a lack of research focusing on Asian countries.

Therefore, this study aims to analyze whether AUD independently affects SI among the residents of Chungnam, who have shown high-risk alcohol consumption rates [21] and SI rates [20] in Korea, by utilizing Propensity Score Matching (PSM). In studies utilizing secondary data, the lack of internal matching between patient and control groups results in low representativeness of the analysis model. Moreover, due to the difficulty in ascertaining the association between AUD and SI, recent efforts have been made to minimize these limitations through PSM [28,29]. Specifically, we aim to identify vulnerable groups for SI among the AUD group and provide basic data for policy and institutional measures for community programs for suicide prevention in the region.

## 2. Methods

### 2.1. Study Sample and Design

This research analyzed the survey data on the 2022 Mental Health Awareness Survey among Chungnam residents conducted in 2022 by the Chungcheongnam-do Mental Health Welfare Center (CHMHC). The CHMHC conducts surveys on the mental health awareness of Chungnam residents aged 20 to 69 living in 16 cities and counties in Chungnam province once a year to use the data as a basic reference for improving the mental health awareness of Chungnam residents and promoting mental health in local communities. The survey selected a total of 1000 valid samples through proportional allocation sampling, considering the standards, age, and regional population composition of the Ministry of the Interior and safety resident registration population statistics as of the end of October 2022. From 5 to 20 November 2022, structured questionnaires were used for both web-based and face-to-face interviews to collect information. 

Among the 1000 respondents, those who did not respond to the survey items on AUD and SI were excluded from the study population. Additionally, respondents who had missing responses to the covariate variables included in the study were further excluded from the study population. Finally, a total of 823 individuals were included in this study.

### 2.2. Independent Variables

#### AUDIT-K

The independent variable in this study is AUD, measured using the Korean version of the Alcohol Use Disorders Identification Test (AUDIT-K) [30]. The questionnaire consists of three items, including frequency and amount of drinking, alcohol dependence, and risky drinking, and it is measured by a total of 10 questions. Each question is scored on a 5-point scale ranging from 0 to 4, and a score of 20 or higher for males and 10 or higher for females on a total score of 40 was used to classify individuals in regard to AUD.

### 2.3. Dependent Variables

#### Suicidal Ideation

The dependent variable in this study was SI. The question asked was “Have you had thoughts of wanting to die in the past 6 months?”. Those who responded, “not at all” were classified as “No”, while those who responded, “once or twice”, “sometimes”, or “often” were classified as “Yes”.

### 2.4. Control Variables

In this study, in order to investigate the association between AUD and SI, we selected socio-economic variables (age, sex, residency region, education level, marital status, household income, occupation type) and health and behavioral factors (anxiety disorder, depressive disorder) as covariates.

#### 2.4.1. Socioeconomic and Demographic Factors

In this study, socioeconomic and demographic variables, including ‘age’, ‘sex’, ‘residence’, ‘education level’, ‘marital status’, ‘household income’, and ‘occupation type’, were selected as social statistical factors to investigate their associations with AUD and SI. ‘Age’ was classified into five categories: ‘20–29 years’, ‘30–39 years’, ‘40–49 years’, ‘50–59 years’, and ‘60–69 years’. ‘Sex’ was classified into ‘male’ and ‘female’, and ‘residency region ‘ was classified into ‘city unit’ and ‘town unit’ areas. ‘Education level’ was classified into ‘less than middle school’, ‘high school’, and ‘college or higher’. ‘Marital status’ was classified into ‘single (including separated and divorced)’ and ‘married’. ‘Family income’ was classified into ‘under 200’, ‘200–500’, and ‘over 500’. Finally, ‘type of occupation’ was defined as ‘economically inactive’, ‘self-employed’, ‘blue-collar’, and ‘white-collar’.

#### 2.4.2. Health Status and Behavioral Factors

The health status and behavioral factors examined in this study were “anxiety disorder” and “depressive disorder”. “Anxiety” was measured using the GAD-7, a self-reported measurement tool developed to screen for patients with anxiety disorders [31]. The tool asked about 7 items related to anxiety or worry, and respondents rated the severity on a scale of 0 (not at all) to 3 (nearly every day). The cut-off point for this scale was 10 points, with a reported sensitivity and specificity of 0.89 and 0.82, respectively [31]. Accordingly, individuals with a total score of 10 or higher were classified as the anxiety group. Additionally, “depressive disorder” was measured using the PHQ-9, a self-reported measurement tool developed to screen for patients with depressive symptoms [32]. The tool asked about 9 items related to major depressive disorder and depression, and respondents rated the severity on a scale of 0 (not at all) to 3 (nearly every day). The cut-off point for this scale was 10 points, with a reported sensitivity and specificity of 0.88 and 0.88, respectively [32]. Accordingly, individuals with a total score of 10 or higher were classified as the depression group.

### 2.5. Analytical Approach and Statistics

In this study, to overcome the limitations of cross-sectional data, we first conducted Propensity Score Matching (PSM). Through this process, we aimed to investigate the pure effect of the independent variable, AUD, on SI in this study.

After controlling for covariates, we used the chi-squared test and matched conditional logistic regression analysis to analyze the difference in distribution between AUD and SI. Data organization and statistical analysis were performed using SAS Ver 9.4 (SAS Institute Inc., Cary, NC, USA), and statistical significance was tested at a 5% significance level.

### 2.6. Propensity Score Matching

PSM is a statistical matching technique that is being used in observational studies to reduce bias. We perform a 1:4 case–control match on the propensity score based on age, sex, residency region, education level, marital status, family income, type of occupation, anxiety disorder, depressive disorder, and AUD. This process ranks best matches first and next-best matches next, creating a hierarchical sequence until no more matches can be made (nearest neighbor matching). The SAS LOGISTIC procedure code is used to create the propensity score [33]. 

Additionally, after PSM matching, if the chi-square tests for all control variables and AUD present *p*-values above 0.05, it indicates that there are no significant differences in any control variables between the AUD group and the non-AUD group. Consequently, this implies a similarity between the AUD and non-AUD groups in regard to age, sex, residency region, education level, marital status, family income, type of occupation, anxiety disorder, and depressive disorder.

## 3. Results

### 3.1. Characteristics of Participants before and after PSM

Table 1 shows the results of the general characteristics of participants before and after 1:4 PSM. Prior to PSM, significant differences were found in the distribution of sociodemographic and health-related factors between the AUD group (13.7%) and the non-AUD group (86.3%). However, after 1:4 PSM, the AUD group (20.0%) and non-AUD (80.0%) showed similar characteristics.

In addition, since the *p*-value for all variables was above 0.05, this indicates that there were no significant differences in the characteristics of variables between the AUD group and the non-AUD group.

### 3.2. General Characteristics of Subjects Included for Analysis after PSM

Table 2 shows the general characteristics of participants according to PSM to analyze the association between AUD and SI. Among the total of 255 participants, 25.5% (N = 65) reported having SI. The AUD group accounted for 20% (N = 51) of the total participants, and the rate of SI in this group was 35.3% (N = 18).

### 3.3. Stratified PS Matched Conditional Logistic of SI

Table 3 presents the results of the analysis that examined the association between AUD and SI after controlling for covariates. The adjusted odds ratio (AOR) for SI in the AUD group was 2.40 times higher than that in the non-AUD group (adjusted odds ratio [AOR]: 2.40, 95% confidence interval [CI], 1.06–5.39).

### 3.4. Stratified PS Matched Conditional Logistic of SI by Sex or Age

**Figure 1:**
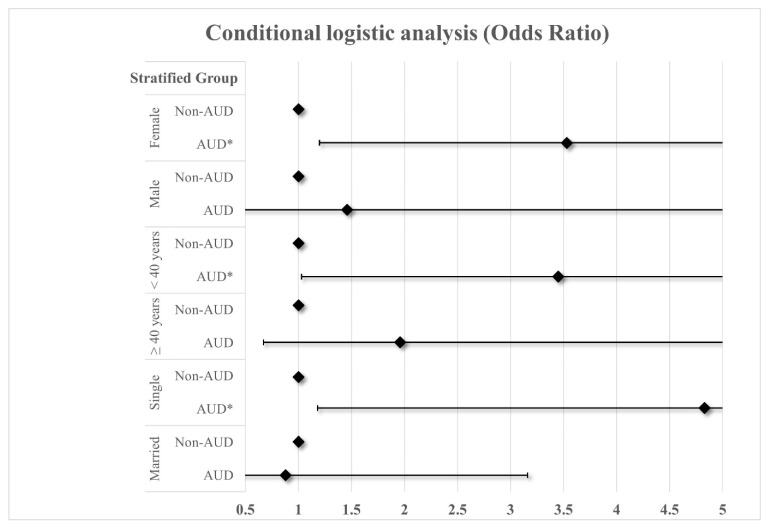
Results of analyzing the association between AUD and SI stratified by sex and age. * <0.05.

Figure 1 shows the results of analyzing the association between AUD and SI stratified by sex and age. In the female group, the odds of SI in the AUD group were 3.53 times higher (AOR: 3.53, 95% CI: 1.20–10.44) compared to the control group, but no significant association was found in the male group. In the group aged under 40, the odds of SI in the AUD group were 3.45 times higher (AOR: 3.45, 95% CI: 1.03–11.55) compared to the control group, but there was no statistically significant association in the group aged 40 or older. In addition, in the single group, the odds of SI in the AUD group were 4.83 times higher (AOR: 4.83, 95% CI: 1.18–19.69) compared to the control group, but no association was found in the married group.

## 4. Discussion

This study aims to provide fundamental data for effective policy development to reduce suicide risk by analyzing the association between AUD and SI using the “2022 Mental Health Awareness Survey” conducted among residents of Chungnam aged 19 and older. 

In summary, the research results are as follows: First, the AUD group had a higher rate of SI compared to the non-AUD group. Second, among women, those under 40 years of age and single in the AUD group had a higher rate of SI compared to the non-AUD group. However, among men, those over 40 years of age and married did not show a significant association between AUD and SI.

According to a report released by the National Institute on Alcohol Abuse and Alcoholism (NIAAA) in the United States [34], AUD is not solely determined by the absolute amount of alcohol consumption, but rather by how much and how often alcohol is consumed in a short period of time (specifically the presence of binge and heavy drinking, which are major risk factors for AUD [34]). Unlike Western countries, where drinking is perceived as a culture that is enjoyed with food, in not only Korea but also Chungnam, the drinking culture is strongly associated with a sense of unity between individuals and group bonding and has a collective meaning. As a result, the frequency of binge and heavy drinking is quite high, and the occurrence of AUD is often not recognized. Compared to other countries, Chungnam shows a high prevalence of AUD [35].

Most of the previous studies have reported that SI caused by AUD is mediated by mental disorders such as anxiety and depression [5,34,36,37]. However, recent studies have shown that acute intoxication in AUD groups [38], in addition to psychological problems, can independently increase the risk of suicide by weakening self-control due to inhibition of the central nervous system [39]. Excessive alcohol consumption has also been found to cause disinhibition in the brain, leading to increased impulsivity and momentary suicidal thoughts [40]. According to a previous study analyzing 13,884 individuals in Australia, AUD groups without any mental disorders had a 1.5 times higher rate of SI compared to the control group [41]. In a 26-year follow-up study of 18,146 individuals in Denmark [42], AUD patients without a previous history of mental illness were reported to have a 5.86 times higher risk of suicide compared to the non-AUD group due to persistent and excessive alcohol consumption leading to serotonin abnormalities, impulsive aggression, and social disconnection.

Meanwhile, the association between AUD and SI can also be explained by individual and socio-economic factors. According to a previous study conducted in France with 516 AUD patients [43], AUD patients experience a decline in their socio-economic status (SES) due to physical impairments such as liver cancer and cirrhosis, which leads to the development of a “Alcohol myopia” phenomenon that creates a pessimistic outlook on their current situation and a sense of poverty, resulting in a rapid increase in SI [43]. In addition, a study analyzing the major causes of SI in 510 AUD patients in Poland [44] found that excessive alcohol consumption stimulates impulsive behavior and cognitive impairment through excessive secretion of mental activity substances, resulting in a higher rate of SI, and community defects and loneliness were also reported as major factors. Based on these previous studies, the results of this study indicating that AUD can independently increase the rate of SI are consistent with previous research.

In addition, this study found a strong association between AUD and SI in the female, under 40, and single groups, which can be explained by several factors. According to previous studies, the rate of women’s social advancement in Chungnam has been steadily increasing, and due to a lenient drinking culture compared to other countries, women’s high-risk drinking rates are also quite high, resulting in a naturally high AUD prevalence rate [27]. In fact, a previous study following up 136 individuals diagnosed with AUD for two years also found that women with AUD had a 3.10 times higher likelihood of SI compared to men due to impulsivity and emotional difficulties caused by alcohol [24]. Additionally, another study [45] reported that women with AUD had a 1.69 times higher likelihood of SI compared to men due to regrets about the past and family stress. Additionally, our findings align with previous research indicating that, in comparison to females, males experiencing lower levels of depression and anxiety are less likely to have suicidal ideation as a result of AUD [24,46]. These factors can be explained by the fact that, even when consuming the same amount of alcohol, women with AUD have a higher vulnerability to the physical effects of alcohol due to rapid absorption and less alcohol dehydrogenase compared to men [47].

In addition, the stronger association between AUD and SI in young age groups can be explained through their overall alcohol consumption and healthcare behavior. Young adults generally have higher frequency and intensity of alcohol consumption compared to middle-aged adults [48], and they are at a higher risk of developing severe AUD due to sustained high-intensity noise exposure in a state of low AUD diagnosis [49]. Excessive alcohol consumption can lead to problems with the endocrine system and other issues, which should be managed through regular medical visits. However, due to high levels of subjectivity regarding personal health and low rates of medical visits, these problems can lead to SI [24]. Meanwhile, one previous study reported that suicidal ideation in the AUD group decreases with age [50]. However, the specific cause could be attributed to differences in the intensity of depression, anxiety symptoms, and alcohol consumption between younger and older age groups.

In the case of single individuals, the lack of factors that can restrain alcohol consumption within the home, along with physical impairments and cognitive decline resulting from chronic alcohol use, can act as impulsive risk factors for suicide, leading to an increase in SI [51,52]. In fact, a previous study conducted in Denmark reported that single individuals with AUD had a 1.45 times higher risk of SI compared to the married group [42]. Also, according to the concept of ”the protective effect of marriage” [53,54], overall, the single group exhibited persistent and excessive drinking behaviors compared to the married group.

As such, despite the need for continuous management of AUD, according to a report by the South Korean Ministry of Health and Welfare, the treatment rate for AUD is 8.1% [55], which is lower than the 11.8% in the United States [27]. Despite the fact that the prevalence of AUD is highest among young people, the utilization rate of treatment services is highest among those in their 40s and 50s, indicating that appropriate treatment services are not being provided at the right time [56]. In addition, it has been reported that in Korea, where the duration of illness is over 10 years and the severity is high, AUD diagnosis and treatment are difficult to cure, and inadequate management systems after discharge lead to social problems such as relapse, emergency illness, and suicide [55]. Meanwhile, in Germany, comprehensive management services for AUD, including education, screening, diagnosis, and treatment, are provided through community care centers, and intensive management services are provided to patients who develop complications and mental illnesses [57]. Based on this, Germany’s annual alcohol consumption rate is 79.4%, the highest in Europe, but the prevalence of AUD is 6.8%, the lowest level, indicating that effective management is being implemented [58].

The limitations of this study are as follows: First, since this study was conducted through online-based surveys, there is a risk of selection bias. Second, the research data used in the analysis are subject to subjective bias due to the mixed opinions of the respondents. Therefore, it is important to acknowledge the objectivity limitations of self-reported data and understand the research results accordingly. Third, since the study participants were residents of the Chungnam province, it is difficult to generalize the results to the entire population of Korea. Therefore, future research should aim to elucidate the association between AUD and SI, and it would be beneficial to not only consider the cultural differences but also explore the association on a national level. Despite the limitations mentioned above, this study has several strengths. First, the use of PSM analysis allows for the verification of the pure effect of AUD by equalizing socio-economic and health-related factors between the AUD and control groups. Second, analyzing residents of Chungnam known for its high rates of risky alcohol consumption and intentional self-harm, provides evidence that can be used as a basis for developing tailored regional policies. Third, the analysis of the association between low awareness of AUD and suicide risk in South Korea is a significant strength of this study.

## 5. Conclusions

This study investigated the association between AUD and SI using the ‘2022 Mental Health Awareness Survey’ conducted on adults aged 19 years and over residing in Chungcheongnam-do.

The results of the study showed that the AUD group had a higher rate of SI compared to the non-AUD group, particularly among females, those under 40 years of age, and single individuals, Strong associations were found within these groups. 

This study identified vulnerable groups with SI in the Chungnam region, which exhibits a high rate of risky drinking in South Korea. In addition, through various previous studies, the importance of community-based management policies for AUD groups was emphasized, highlighting the need for treatment policies to prevent suicide. Based on this, it is expected that the suicide rate among AUD groups residing in the Chungnam region will decrease.

However, since this study was conducted as a cross-sectional study, future longitudinal analyses are necessary to determine the causal relationship between AUD and SI.

## Figures and Tables

**Table 1:** Characteristics of participants before and after PSM.

Variables	Before PSM							After PSM						
	Total		Non-AUD		AUD		*p*-Value	Total		Non-AUD		AUD		*p*-Value
	N	%	N	%	N	%		N	%	N	%	N	%	
Total	823	100.0	710	86.3	113	13.7		255	100.0	204	80.0	51	20.0	
Sex							<0.0001							0.750
Male	390	47.4	312	80.0	78	20.0		150	58.8	119	79.3	31	20.7	
Female	433	52.6	398	91.9	35	8.1		105	41.2	85	81.0	20	19.0	
Age							<0.0001							0.987
20–29	135	16.4	96	71.1	39	28.9		54	21.1	42	77.8	12	22.2	
30–39	155	18.8	130	83.9	25	16.1		56	22.0	46	82.1	10	17.9	
40–49	193	23.5	158	81.9	35	18.1		79	31.0	63	79.7	16	20.3	
50–59	179	21.7	172	96.1	7	3.9		30	11.8	24	80.0	6	20.0	
60–69	161	19.6	154	95.7	7	4.3		36	14.1	29	80.6	7	19.4	
Residency region							0.051							0.054
City unit	666	80.9	567	85.1	99	14.9		217	85.1	169	77.9	48	22.1	
Town unit	157	19.1	143	91.1	14	8.9		38	14.9	35	92.1	3	7.9	
Marital status							<0.0001							0.095
Single (including separated, divorced)	249	30.3	193	77.5	56	22.5		99	38.8	74	74.7	25	25.3	
Married	574	69.7	517	90.1	57	9.9		156	61.2	130	83.3	26	16.7	
Family income							0.574							0.297
<200	96	11.7	76	79.2	20	20.8		36	14.1	32	88.9	4	11.1	
200–500	416	50.5	358	86.1	58	13.9		127	49.8	98	77.2	29	22.8	
≥500	311	37.8	276	88.7	35	11.3		92	36.1	74	80.4	18	19.6	
Education level							0.099							0.546
≤Middle school	73	8.9	69	94.5	4	5.5		18	7.1	16	88.9	2	11.1	
High school	314	38.2	269	85.7	45	14.3		99	38.8	77	77.8	22	22.2	
≥College	436	53.0	372	85.3	64	14.7		138	54.1	111	80.4	27	19.6	
Type of occupation							0.162							0.913
Economically inactive	55	6.7	46	83.6	9	16.4		20	7.8	17	85.0	3	15.0	
Self-employment	119	14.5	109	91.6	10	8.4		26	10.2	20	76.9	6	23.1	
Blue collar	322	39.1	281	87.3	41	12.7		108	42.4	87	80.6	21	19.4	
White collar	327	39.7	274	83.8	53	16.2		101	39.6	80	79.2	21	20.8	
Anxiety disorder							<0.0001							0.679
No	713	86.6	640	89.8	73	10.2		229	89.8	184	80.3	45	19.7	
Yes	110	13.4	70	63.6	40	36.4		26	10.2	20	76.9	6	23.1	
Depression disorder							<0.0001							0.494
No	687	83.5	626	91.1	61	8.9		234	91.8	186	79.5	48	20.5	
Yes	136	16.5	84	61.8	52	38.2		21	8.2	18	85.7	3	14.3	

PSM: Propensity Score Matching/AUD: Alcohol Use Disorder.

**Table 2:** General characteristics of subjects included for analysis after PSM.

Variables	Suicidal Ideation						
	Total		No		Yes		*p*-Value
	N	%	N	%	N	%	
Total	255	100.0	190	74.5	65	25.5	
AUDIT-K							0.073
Non-AUD	204	80.0	157	77.0	47	23.0	
AUD	51	20.0	33	64.7	18	35.3	
Sex							0.514
Male	150	58.8	114	76.0	36	24.0	
Female	105	41.2	76	72.4	29	27.6	
Age							0.000
20–29	54	21.2	44	81.5	10	18.5	
30–39	56	22.0	31	55.4	25	44.6	
40–49	79	31.0	56	70.9	23	29.1	
50–59	30	11.8	28	93.3	2	6.7	
60–69	36	14.1	31	86.1	5	13.9	
Residency region							0.899
City unit	217	85.1	162	74.7	55	25.3	
Town unit	38	14.9	28	73.7	10	26.3	
Marital status							0.010
Single (including separated, divorced)	99	38.8	65	65.7	34	34.3	
Married	156	61.2	125	80.1	31	19.9	
Family income							0.017
<200	36	14.1	24	66.7	12	33.3	
200–500	127	49.8	88	69.3	39	30.7	
≥500	92	36.1	78	84.8	14	15.2	
Education level							0.458
≤Middle school	18	7.1	13	72.2	5	27.8	
High school	99	38.8	78	78.8	21	21.2	
≥College	138	54.1	99	71.7	39	28.3	
Type of occupation							0.119
Economically inactive	20	7.8	12	60.0	8	40.0	
Self-employment	26	10.2	23	88.5	3	11.5	
Blue collar	108	42.4	77	71.3	31	28.7	
White collar	101	39.6	78	77.2	23	22.8	
Anxiety disorder							<0.0001
No	229	89.8	182	79.5	47	20.5	
Yes	26	10.2	8	30.8	18	69.2	
Depression disorder							<0.0001
No	234	91.8	185	79.1	49	20.9	
Yes	21	8.2	5	23.8	16	76.2	

PSM: Propensity Score Matching/AUD: Alcohol Use Disorder.

**Table 3:** Matched conditional logistic regression of suicidal ideation.

Variables	Suicidal Ideation			
	Crude OR	95% CI	AOR	95% CI
AUDIT-K				
Non-AUD	1.00		1.00	
AUD	1.82	(0.94–3.53)	2.40 *	(1.10–5.22)
Sex				
Male	1.00		1.00	
Female	1.21	(0.68–2.13)	0.81	(0.35–1.87)
Age				
20–29	1.00		1.00	
30–39	3.55 **	(1.49–8.43)	2.43	(0.83–7.15)
40–49	1.81	(0.78–4.19)	2.25	(0.77–6.57)
50–59	0.31	(0.06–1.54)	0.58	(0.09–3.55)
60–69	0.71	(0.22–2.28)	0.55	(0.08–3.66)
Residency region				
City unit	1.00		1.00	
Town unit	1.05	(0.48–2.30)	1.22	(0.48–3.11)
Marital status				
Single (including separated, divorced)	2.11 *	(1.19–3.74)	1.50	(0.63–3.62)
Married	1.00		1.00	
Family income				
<200	2.79 *	(1.14–6.83)	1.96	(0.56–6.80)
200–500	2.47 **	(1.25–4.89)	2.09	(0.93–4.69)
≥500	1.00		1.00	
Education level				
≤Middle school	0.98	(0.33–2.92)	3.03	(0.47–19.62)
High school	0.68	(0.37–1.26)	1.00	(0.48–2.10)
≥College	1.00		1.00	
Type of occupation				
Economically inactive	2.26	(0.83–6.20)	1.56	(0.46–5.35)
Self-employment	0.44	(0.12–1.61)	0.78	(0.16–3.79)
Blue collar	1.37	(0.73–2.55)	1.16	(0.54–2.46)
White collar	1.00		1.00	
Anxiety disorder				
No	1.00		1.00	
Yes	8.71 ***	(3.57–21.26)	2.73	(0.89–8.41)
Depression disorder				
No	1.00		1.00	
Yes	12.08 ***	(4.22–34.61)	5.30 *	(1.33–21.09)

* *p* < 0.05. ** *p* < 0.01. *** *p* < 0.001.

## Data Availability

Data are contained within the article.
